# Iron and *Acinetobacter baumannii* Biofilm Formation

**DOI:** 10.3390/pathogens3030704

**Published:** 2014-08-18

**Authors:** Valentina Gentile, Emanuela Frangipani, Carlo Bonchi, Fabrizia Minandri, Federica Runci, Paolo Visca

**Affiliations:** Department of Sciences, Roma Tre University, Viale Marconi 446, 00146 Rome, Italy; E-Mails: valentina.gentile@uniroma3.it (V.G.); emanuela.frangipani@uniroma3.it (E.F.); carlo.bonchi@uniroma3.it (C.B.); fabrizia.minandri@uniroma3.it (F.M.); federica.run@gmail.com (F.R.)

**Keywords:** *Acinetobacter baumannii*, biofilm, chelator, deferasirox, deferiprone, deferoxamine, dipyridyl, iron, transferrin

## Abstract

*Acinetobacter baumannii* is an emerging nosocomial pathogen, responsible for infection outbreaks worldwide. The pathogenicity of this bacterium is mainly due to its multidrug-resistance and ability to form biofilm on abiotic surfaces, which facilitate long-term persistence in the hospital setting. Given the crucial role of iron in *A. baumannii* nutrition and pathogenicity, iron metabolism has been considered as a possible target for chelation-based antibacterial chemotherapy. In this study, we investigated the effect of iron restriction on *A. baumannii* growth and biofilm formation using different iron chelators and culture conditions. We report substantial inter-strain variability and growth medium-dependence for biofilm formation by *A. baumannii* isolates from veterinary and clinical sources. Neither planktonic nor biofilm growth of *A. baumannii* was affected by exogenous chelators. Biofilm formation was either stimulated by iron or not responsive to iron in the majority of isolates tested, indicating that iron starvation is not sensed as an overall biofilm-inducing stimulus by *A. baumannii*. The impressive iron withholding capacity of this bacterium should be taken into account for future development of chelation-based antimicrobial and anti-biofilm therapies.

## 1. Introduction

*Acinetobacter baumannii* has emerged worldwide as a leading cause of hospital-acquired infections, especially among severely ill patients in intensive care units (ICUs) [1]. Although *A. baumannii* was initially regarded to as a low-grade pathogen, evidence has been accumulated suggesting that *A. baumannii* infections are associated with increased mortality in critically ill patients [2]. *A. baumannii* causes a broad range of nosocomial infections, including ventilator-associated pneumonia, urinary tract infections, wound infection, bacteremia, endocarditis, meningitis [3], and has recently been associated with very severe community-acquired infections, especially among individuals with predisposing factors in Southern Asia and other tropical regions [4]. *A. baumannii* can also be isolated from veterinary sources, and show common characteristics with strains described in human infection [5].

Tendency to the epidemic spread, resistance to antibiotics and persistence in the hospital setting are hallmarks of *A. baumannii* infection [3]. Successful strains of multidrug-resistant (MDR) *A. baumannii* are notorious for their ability to rapidly spread among hospitalized patients, overcome geographical borders, and become epidemic worldwide [6]. Epidemiologic and population genetics studies indicate that the majority of *A. baumannii* infections are caused by strains belonging to three international clonal lineages (ICLs) [1,3,6]. *A. baumannii* strains belonging to the most widespread ICLs are invariably characterized by an MDR phenotype, which is progressively evolving towards pandrug resistance, thereby challenging the current antimicrobial armamentarium [7,8]. This poses the urgent need for the development of novel treatment strategies to combat infections caused by MDR *A. baumannii* [9].

The capacity of MDR clinical isolates of *A. baumannii* to resist to desiccation and to form biofilms are regarded as crucial factors contributing to the clinical success and persistence of this species in healthcare facilities. *A. baumannii* can survive for up to months on the dry surface of inanimate objects [10,11], enabling transmission of infection for long times under both epidemic and endemic situations [12]. A number of reports have demonstrated that *A. baumannii* can form biofilms on several biotic and abiotic surfaces, providing the bacteria with protection against antibiotic/antiseptic treatment(s) and the host immune defenses *in vivo* (reviewed in [13,14]). Biofilm formation is crucial for several *A. baumannii* infections, since these are often associated with indwelling medical devices, e.g., vascular and urinary catheters, cerebrospinal fluid shunts, and endotracheal tubes [15]. While it is apparent that the capacity to form biofilms is a general phenotypic trait of *A. baumannii*, remarkable differences in the amount of biofilm formed by different strains have been reported, even if belonging to the same clonal lineage or epidemiological cluster [12,15,16,17,18]. A number of environmental factors can influence biofilm formation, including the presence of metal cations [16,19]. Among these, iron represents an essential nutrient for infecting bacteria, and a key determinant in host-pathogen interactions. This is because bacteria must counteract an iron-poor environment during infection, due to iron sequestration by iron carrier and storage proteins of the host and adaptive hypoferremia during infection [20]. *A. baumannii* has evolved an impressive capacity to acquire iron from the host, due to the production of multiple siderophores for Fe(III) transport, combined with uptake specificities for heme and Fe(II) [21,22].

Given the crucial role of iron in *A. baumannii*-host interactions [22,23,24], attention has recently been given to non-antibiotic approaches that target iron metabolism to achieve antibacterial activity, including chelation therapy and use of iron mimetics (reviewed in [9]). Interestingly, it was noted that: (*i*) high concentrations of deferiprone (DFP, (Sigma Aldrich, St. Louis, MO, USA)), a compound used for chelation therapy in humans, inhibited to some extent logarithmic growth of *A. baumannii* ATCC 17978 in a chemically defined medium [25]; (*ii*) gallium, an iron-mimetic drug, suppressed the growth of MDR *A. baumannii* strains both *in vitro* and *in vivo*, acting through disruption of bacterial iron metabolism [25,26,27]; (*iii*) mutants impaired in production of the acinetobactin siderophore show reduced fitness *in vivo* [23]. On the other hand, it was also reported that biofilm formation on plastic by the type strain *A. baumannii* ATCC 19606^T^ was stimulated under conditions of iron scarcity imposed by the addition of the chelator 2,2ʹ-dipyridyl (DIP) [19]. Therefore, the effect of iron availability on both planktonic and biofilm mode of *A. baumannii* growth deserves more in-depth investigation.

In this report, strains and optimal growth conditions for the generation *A. baumannii* biofilms were preliminarily established. Then, the role of iron in *A. baumannii* biofilm formation was investigated. Lastly, the activity of a new therapeutic iron chelator was assessed in search for inhibitory drugs that could be repurposed as adjuvant antimicrobials in the treatment of biofilm-based *A. baumannii* infections.

## 2. Results and Discussion

### 2.1. Definition of Culture Conditions for *A. baumannii* Biofilm Formation

**Figure 1:**
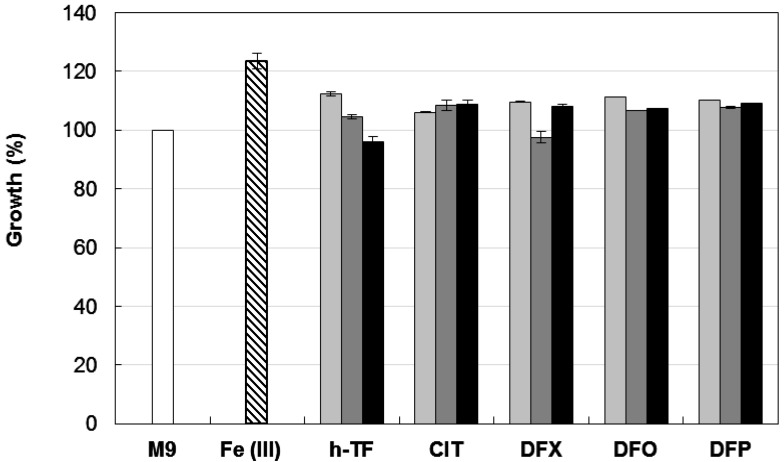
Effect of different iron chelators on planktonic growth of *A. baumannii* ATCC 17978. Bacteria were grown for 48 h at 37 °C in 96-wells microtiter plates containing 100 μL M9 supplemented with the indicated iron chelator at different concentrations: 32 μM (light grey bars), 128 μM (dark grey bars) or 128 μM chelator + 100 μM DIP (black bars). Growth was measured as OD_600_ and expressed as percentage relative to the untreated control (*i.e.*, OD_600_ in M9). The average of the OD_600_ in control M9 was 0.318 ± 0.008 and represents 100% of growth (white bar). Relative growth in M9 supplemented with 100 μM FeCl_3_ is reported (striped bar). Data represent the average of three independent experiments ± standard deviation. h-TF, tranferrin; CIT, citrate; DFX, deferasirox; DFO, desferrioxamine; DFP, deferiprone.

Biofilm formation is a multifactorial phenotype [13,14], and in *A. baumannii* it can be modulated by iron availability, carbon sources, growth temperature, and different expression levels of adhesive and cell-aggregating factors [13,14,16,18,19,28]. Therefore, as a preliminary step to the investigation of the effect of iron on *A. baumannii* biofilm formation, we determined the growth response of the reference strain *A. baumannii* ATCC 17978 [25,29] to iron restriction imposed by different chelators in M9 minimal medium [30] containing 20 mM sodium succinate as the carbon source [26]. In line with previous observations [25], *A. baumannii* ATCC 17978 showed an impressive ability to multiply under conditions of iron deficiency, as those imposed by the addition of up to 128 µM human apo-transferrin (h-TF (Sigma Aldrich)), trisodium citrate (CIT (Sigma Aldrich)), desferrioxamine (DFO (Ciba Geigy, Origgio, Italy)), deferasirox (DFX (Novartis, Basel, Switzerland)) and DFP (Figure 1).

None of the tested chelators reduced *A. baumannii* ATCC 17978 growth yields at 48 h, even when 100 µM DIP (a chelator of the intracellular Fe(II) pool) was added to further reduce iron availability. As expected, growth in M9 was stimulated by *ca.* 25% in the presence of 100 µM FeCl_3_. A similar resistance to exogenously supplied chelators in M9 was also observed for strains AYE [31] and ACICU [32], representatives for ICL-I and ICL-II, respectively (data not shown). These data can be explained by the presence in *A. baumannii* of very efficient iron uptake systems [11,21], capable of counteracting the iron withholding capacity of exogenously added chelators. The observation that DFX, DFO, and DFP do not stimulate bacterial growth in the presence of DIP (a chelator of the intracellular Fe(II) pool) suggests that these chelators are unlikely to serve as an iron source for *A. baumannii*.

**Figure 2:**
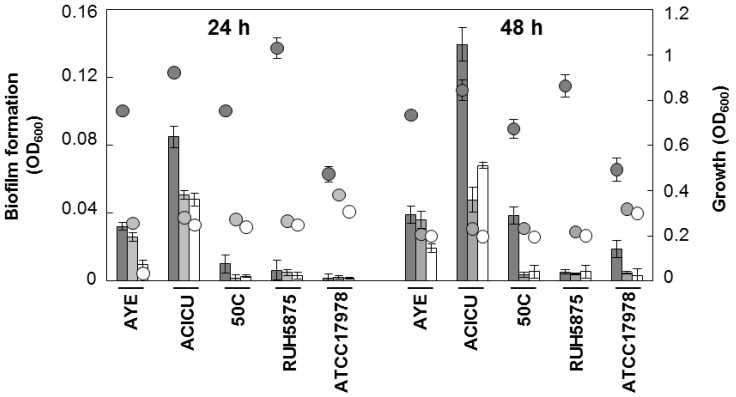
Growth and biofilm formation by selected *A. baumannii* strains in different iron-poor media. Bacterial cells were inoculated at OD_600_ of 0.01 in 100 μL of the different growth media, dispensed in a 96-wells microtiter plate, and grown at 37 °C without shaking for 24 and 48 h. Growth (circles) was measured spectrophotometrically (OD_600_) and biofilm formation (bars) was evaluated using the CV staining assay [36]. Dark grey, TSBD; light grey M9; white M9 supplemented with 100 μM DIP. Data represent the average of three independent experiments ± standard deviation.

Next, the inter-strain variability and the growth medium-dependence of biofilm formation was investigated. Five well-characterized *A. baumannii* strains (AYE, representative for ICL-I [31]; ACICU, representative for ICL-II [32]; 50C, ICL-II pandrug resistant isolate [33]; RUH5875, prototypic strain for ICL-III [34]; ATCC 17978 [29]] were grown in three iron-poor media [M9, M9 supplemented with 100 μM DIP, and Chelex-100-treated Tryptic Soy Broth dialysate, TSBD [35]) in order to determine both growth and biofilm levels at 24 and 48 h. Quantitative estimation of the bacterial biomass in biofilms was assessed in 96-well polystyrene microtiter plates (BD Falcon, Milano, Italy), using the crystal-violet (CV) staining method [36]. There was a wide range of variation in growth and biofilm levels between *A. baumannii*, depending on strains and culture media, though for some strains moderate correlation was observed between growth yields and biofilm levels (Figure 2).

Remarkably, all strains produced more abundant biofilm in TSBD than in the other iron-poor media, and biofilm levels in strain ACICU were significantly higher (*p* < 0.05 in the student’s *t*-test) than all the other strains tested (Figure 2). These findings corroborate the notion that biofilm levels in *A. baumannii* can vary even between closely related isolates (e.g., strains ACICU and 50C belonging to the same genetic cluster according to ref. [33]), and that different media have a profound impact on biofilm yields [15,16,17,18].

**Figure 3:**
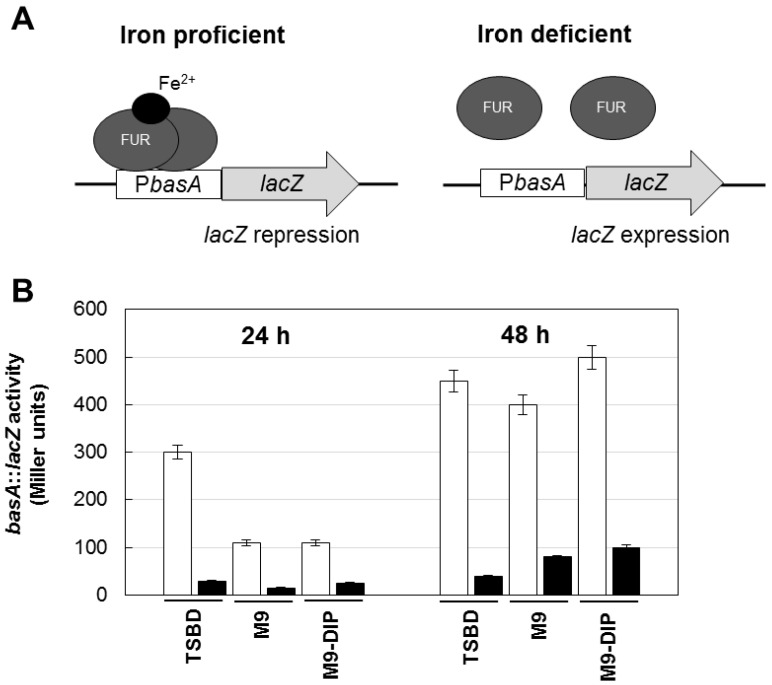
Regulatory mechanism and activity of the *basA::lacZ* iron biosensor in the reference *A. baumannii* strain ATCC 17978. (A) Schematic of the regulatory mechanism the *basA::lacZ* iron-regulated transcriptional fusion carried by plasmid pMP220::P*_basA_* [26]. Under iron proficient conditions (left), the Fur repressor protein binds the P*_basA_* promoter and inhibits β–galactosidase (LacZ) expression; under iron deficient conditions Fur repression is relieved and the LacZ enzyme is expressed. (B) Activity of the *basA::lacZ* iron-regulated fusion in *A. baumannii* ATCC 17978 grown for 24 and 48 h in different media, as indicated, in the absence (white bars) or presence (black bars) of 100 μM FeCl_3_. Data are the means (±standard deviations (SD)) of triplicate experiments.

To rule out the possibility that differences in biofilm levels between TSBD and M9 or M9 plus DIP were due to different iron content of these media, an iron biosensor consisting of the Fur-controlled *basA* promoter fused to the reporter *lacZ* gene [26] was used as a probe to determine the intracellular iron level in *A. baumannii* ATCC17978. Since the Fur repressor protein acts as an iron sensor, the activity of the Fur-controlled *basA* promoter provides an indirect estimate of the intracellular iron levels of bacteria grown in the different media. The β–galactosidase (LacZ) expression was higher in TSBD than in M9 or M9 plus DIP (Figure 3), and it was invariably repressed by iron, indicating that TSBD is sensed by *A. baumannii* as an iron-poor medium.

**Figure 4:**
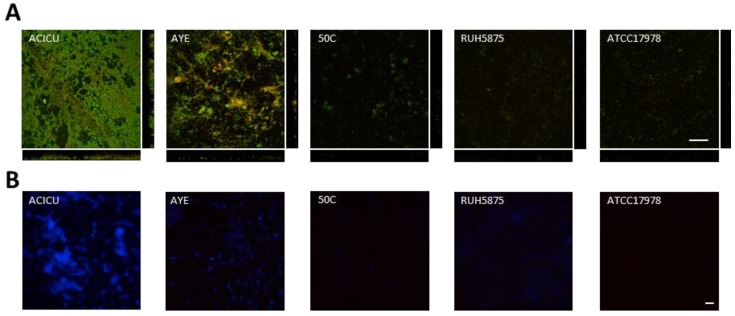
Seven-days biofilm of selected *A. baumannii* strains grown in TSBD. (**A**) Confocal microscope images (x-y plane and side view) of *A. baumannii* biofilms stained with acridine orange, a fluorescent dye which labels double-stranded nucleic acids (prevalently DNA) in green, and single-stranded nucleic acids (prevalently RNA) in red. (**B**) *A. baumannii* biofilms stained with the calcofluor white for exopolysaccharide labelling [19,28], and analyzed by fluorescence microscopy. Scale bar: 50 μm.

To visualize differences in biofilm structure among the five representative *A. baumannii* strains, biofilm formation on glass slides was monitored during seven days growth in TSBD by means of confocal microscopy, according to a previously described procedure [37] (Figure 4). High biofilm levels with formation of large cellular aggregates were observed for *A. baumannii* ACICU, and to a much lesser extent for the other strains (Figure 4A). Interestingly, *A. baumannii* biofilm cells were found to be embedded in a blue fluorescent material upon staining with calcofluor white (Figure 4B). In line with previous findings [18,29], this observation denotes the presence of exopolysaccharides in the matrix of *A. baumannii* biofilms, whose levels appear to be consistent with to the amount of biofilm formed in 96-well polystyrene microtiter plates (Figure 2).

Based on the above results, TSBD was considered as suitable iron-depleted medium that would allow robust biofilm formation and an easier evaluation of the effect of iron on this process. This is because the high biofilm levels achieved by *A. baumannii* in TSBD would facilitate the detection of biofilm variations in response to iron levels. Moreover, the high peptide content and balanced formula of TSBD (35, see also [38]) make it more similar to a biological fluid than the M9 mineral salt medium.

### 2.2. Effect of Iron Levels on Biofilm Formation by a Collection of Diverse A. baumannii Isolates

**Figure 5:**
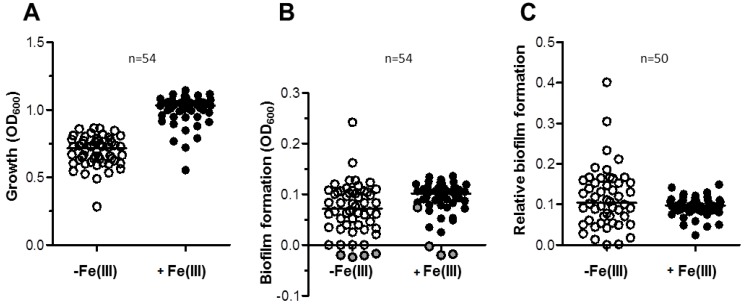
Growth and biofilm formation in a representative collection of 54 *A. baumannii* strains from clinical and veterinary sources. (**A**) Growth of 54 *A. baumanni* strains for 48 h in 96-wells microtiter plates containing 100 µL TSBD supplemented (black circles) or not (white circles) with 100 μM FeCl_3_, as indicated. (**B**) Absolute values of biofilm formation by the same isolates shown in panel A, evaluated by the CV staining assay (OD_600_). Grey circles (B) represent the values for strains that in either or both conditions yielded negative biofilm values, and were excluded from calculations in panel C. (**C**) Relative values of biofilm formation (Biofilm formation (OD_600_)/Growth (OD_600_)) for a subset of 50 biofilm-producing isolates. The line bar represents the median value for each group. Values for each strain are the average of three independent experiments.

We investigated the effect of iron on biofilm formation in a representative collection of 54 *A. baumannii* strains (Table S1 in Supplementary Material), including 27 isolates from veterinary sources (67% MDR) and 27 isolates from clinical sources (96% MDR). Isolates were selected so to maximize diversity, as inferred by Random Amplified Polymorphic DNA fingerprints with primer DAF4 ([21,33]; data not shown), and represent the most widespread ICLs and some emerging lineages (Table S1 in Supplementary Material). High growth yields were observed for almost all isolates in TSBD (median OD_600_ = 0.716), which were significantly increased by the addition of 100 µM FeCl_3_ (median OD_600_ = 1.031), consistent with iron being a nutritionally-limiting factor in TSBD (Figure 5A). Remarkably, biofilm formation was more abundant in FeCl_3_-supplemented TSBD (median OD_600_ = 0.102) than in TSBD (median OD_600_ = 0.071) (Figure 5B). After having excluded from the analysis 4 biofilm non-producing isolates (namely, 4297, 196-1, 82D, RUH5875, see Table S1), the normalization of biofilm formation by growth yields resulted in minor differences between the two growth conditions (median values were 0.104 and 0.098 for the iron-limited and iron-rich condition, Figure 5C). This result is due to somehow opposite responses of *A. baumannii* isolates to iron starvation (*i.e.*, TSBD *vs.* FeCl_3_-supplemented TSBD); in 21 isolates (42%) biofilm production was significantly enhanced by iron deficiency, in 12 (24%) it was significantly reduced, and in 17 (34%) iron had no effect on biofilm formation (significance in the Student’s *t*-test was set at *p* < 0.05). Although stimulation of biofilm formation in response to iron-limited growth was observed for a minority (42%) of *A. baumannii* isolates, this may have relevant medical implications, since transition of these isolates from planktonic to biofilm-growing cells could be favored *in vivo*, where infecting bacteria are normally challenged with iron shortage. However, this behavior cannot be generalized, since biofilm production was either unchanged or even inhibited by iron deficiency in the majority (66%) of the isolates.

### 2.3. Effect of Deferasirox on A. baumannii Biofilm Formation

We showed that planktonic *A. baumannii* has an impressive ability to grow in the presence of exogenously added therapeutic chelators DFP, DFO, and DFX (Figure 1), and previous data indicate only modest inhibition of *A. baumannii* growth at high DFP concentrations (*ca.* 200 µM, ref. [25]). To gain further insight into the effect of iron withholding on *A. baumannii* biofilm formation, we examined the effect of DFX, a newly developed orally active Fe(III) chelator, on our collection of 50 biofilm-producing isolates. DFX is a synthetic compound with high affinity and specificity for Fe(III) (logβ_2_ = 36.9 according to ref. [39]), and is unlikely to serve as an iron carrier to *A. baumannii* based on growth assays (Figure 1). It was successfully used in combination therapy against murine staphylococcemia [40], and in treatment of invasive fungal infections [41].

**Figure 6:**
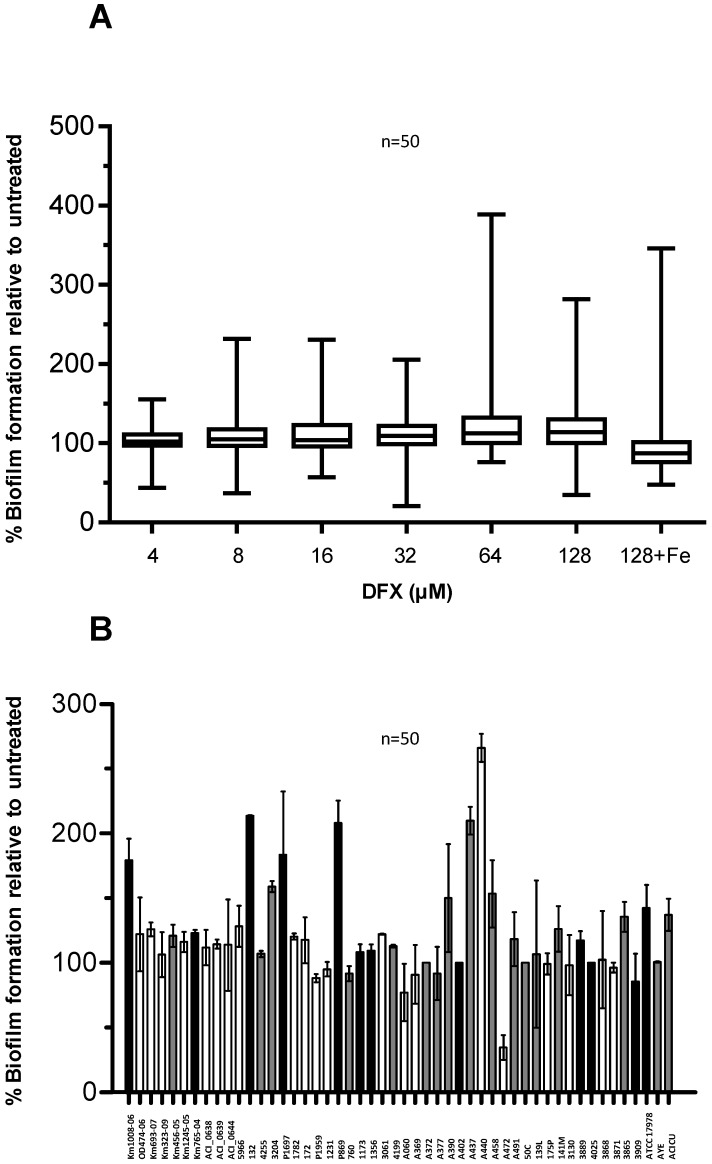
Effect of DFX on *A. baumannii* biofilm formation. (**A**) *A. baumannii* strains were grown statically for 48 h in microtiter plates containing 100 μL TSBD supplemented with DFX at indicated concentrations, or 128 µM DFX plus 100 µM FeCl_3_. Biofilm formation (OD_600_ in the CV staining assay) was normalized by the growth yield (OD_600_ of the culture) and expressed as percentage relative to the DFX-untreated control (TSBD). Boxes represent medians, second and third interquartiles; whiskers represent range of 50 isolates tested. (**B**) Relative biofilm levels produced by individual isolates in presence of 128 µM DFX, expressed as % of the untreated control in TSBD. With reference to Figure 5C, the bar filling denotes: isolates in which biofilm production was significantly enhanced by iron deficiency (white, 21 isolates), or significantly reduced (black, 12 isolates), or in which iron had no effect on biofilm formation (grey, 17 isolates). In both panels data represent the average of three independent experiments.

Here, biofilm formation was tested in a DFX concentration range 4–128 µM, in order to match the DFX plasma levels achievable during treatment of iron overload in humans [42]. Notably, biofilm formation by most of the isolates was not significantly affected by DFX up to 128 µM, either in TSBD or in TSBD plus 100 µM FeCl_3_ (Figure 6A).

No obvious correlation could be observed between the biofilm response to iron starvation in TSBD (see Figure 5 and associated text) and in the presence of 128 µM DFX (Figure 6B). The observation that a minority of isolates (*i.e.*, Km1008-06, 132, P1697, P869, A472, and ATCC17978) showed opposite responses to iron starvation induced by TSBD with and without iron (Figure 5), compared with TSBD and TSBD plus DFX (Figure 6B), suggests that either DFX exerts iron chelation-independent effects, or that the iron deficiency threshold that determines the biofilm response in *A. baumannii* can vary for these isolates. Apart from this, the ability to generate biofilms is scarcely influenced by the presence of DFX for most *A. baumannii* isolates, indicating that, at least *in vitro*, this therapeutic chelator is incapable of overcoming the iron withholding capacity of *A. baumannii* biofilms.

## 3. Experimental Section

### 3.1. Bacterial Strains and Growth Media

Relevant characteristics of the 54 *A. baumannii* strains used in this study are provided in Table S1 in the Supplementary Material. The collection includes representative strains for ICLs I, II, and III, namely AYE (ICL-I) [43], ACICU (ICL-II) [32], and RUH 5875 (ICL-III). Strain ATCC 17978 is a well-characterized clinical isolate dated 1950s and showing moderate antibiotic resistance [29]. Strain 50C is a pandrug resistant clinical isolate [33,44]. Other clinical and veterinary isolates were provided as part of the collections from various European laboratories [5,21,45,46,47,48]. Iron-poor culture media used in this study were the M9 minimal medium [30] supplemented with 20 mM sodium succinate as the carbon source, and TSBD, a Chelex 100-treated Trypticase Soy Broth dialysate [35]. When required, media were supplemented with either 100 µM DIP or 100 µM FeCl_3_.

### 3.2. Chemicals

The chemicals used in this study were deferiprone (DFP, (Sigma Aldrich)), tri-sodium citrate [CIT, (Sigma Aldrich), desferrioxamine (DFO, (Ciba Geigy)); human apo-transferrin (h-TF, iron content ≤ 0.005%, (Sigma Aldrich)), and deferasirox (DFX, (Novartis)).

### 3.3. Growth Inhibitory Activity of Iron-Chelators

The activity of the iron chelators on bacterial growth was tested in 96-well microtiter plates (BD Falcon) containing increasing concentrations (4–128 μM) of iron chelators. Plates were inoculated at OD_600_ of 0.01 in a 100 μL final volume of M9 supplemented or not with 100 µM DIP at the highest iron-chelator concentration tested, or 100 µM FeCl_3_, and incubated at 37 °C for 48 h with moderate shaking (100 r.p.m.). Spectrophotometric readings were performed in a Wallac 1420 Victor3V multilabel plate reader (Perkin Elmer, Milano, Italy).

### 3.4. Biofilm Formation

Biofilm formation was measured according to the microtiter plate assay [36]. Briefly, bacterial cells were inoculated at OD_600_ of 0.01 in 100 μL of medium and grown at 37 °C for up to 48 h in 96-wells microtiter plates without shaking. Planktonic cells were removed and the attached cells were gently washed three times with sterile PBS, air dried, and stained with 150 µL of 0.1% CV water solution for 20 min. The wells were gently washed four times with distilled water, and the surface-associated dye was eluted in 200 µL of 95% ethanol. The OD_600_ of the eluate was measured in a Wallac 1420 Victor3V multilabel plate reader (Perkin Elmer).

### 3.5. Biofilm Inhibition

To investigate the effect of DFX on biofilm formation, fifty biofilm-producing *A. baumannii* strains were inoculated (OD_600_ of 0.01) into 96-well microtiter plates containing 100 μL TSBD supplemented with increasing DFX concentrations (4 to 128 µM) or 128 µM DFX plus 100 µM FeCl_3_ The assay was performed as described above.

### 3.6. Microscopy Analysis

For microscopic visualization of *A. baumannii* biofilms, strains were grown in an 8-well chamber slide as previously described [37]. Briefly, bacteria were inoculated at OD_600_ of 0.01 in 200 μL of TSBD and incubated at 37 °C for 48 h to allow the adhesion of the bacterial cells on the glass surface. To maintain bacterial viability, the medium was changed every 24 h until the seventh day. To visualize biofilms structure, *A. baumannii* biofilms were stained with the acridine orange (0.1% water solution), a fluorescent dye, which labels double-stranded nucleic acids (prevalently DNA) in green, and single-stranded nucleic acids (prevalently RNA) in red, and examined using Leica TCS SP5 confocal microscope. For detection of matrix exopolysaccharides, samples were stained with calcofluor white (Fluka) and analyzed with an epifluorescence microscope. The Image J software [49] was used for image analysis.

### 3.7. β-Galactosidase Activity Assay

The *basA* promoter was cloned upstream of the *lacZ* reporter gene in plasmid pMP220::P*basA* (carrying the tetracycline-resistance (Tc^R^) determinant) as previously described [26]. For reporter gene activity measurements, *A. baumannii* ATCC 17978 (Tc^S^) was transformed with P*basA::lacZ* (Tc^R^) and grown for 16 h at 37 °C in M9 medium supplemented with 10 µg/mL Tc. Cultures were then appropriately diluted in TSBD, M9 and M9 supplemented with 100 µM DIP, with or without 100 µM FeCl_3_ to reach an initial cell concentration corresponding to OD_600_ ~ 0.01 and incubated at 37 °C with vigorous shaking. The β-galactosidase (LacZ) activity expressed by *A. baumannii* ATCC 17978 (pMP220::P*basA*) after 24 and 48 h growth was determined spectrophotometrically on toluene/SDS-permeabilized cells using *o*-nitrophenyl-β-d-galactopyranoside as the substrate, and expressed in Miller units [50].

Miller units = 1,000 × [OD_420_ – (1.75 × OD_550_)] / Volume (ml) × Time (min) × OD_600_

## 4. Conclusions

The formation and maturation of *A. baumannii* biofilms depend on the complex interplay of many environmental and cell-associated factors [13,14]. In this study, attention has been focused on the role of iron, since this metal is essential for bacterial nutrition and virulence [22,23,24], and plays a central role in host defense from bacterial infection [20]. In agreement with a previous study [25], we showed that planktonic *A. baumannii* cells can overcome iron restriction imposed by a variety of exogenous chelators, likely due to the presence in this species of multiple iron scavenging systems [21,22]. Then, we observed relevant differences in biofilm levels depending on *A. baumannii* strain and growth medium, and established suitable conditions for testing the effect of iron on biofilm formation. The two most relevant findings of these experiments were: (*i*) the strong influence of medium composition on biofilm yields; (*ii*) the high variability in biofilm levels produced by *A. baumannii* strains of clinical and veterinary origin, irrespective of their genetic relatedness or epidemic potential; (*iii*) the strain-dependent response of *A. baumannii* biofilms to iron scarcity. Since biofilm formation was either stimulated by iron or not responsive to this metal in the majority of strains tested, we conclude that iron starvation is not sensed as an overall biofilm-inducing stimulus by *A. baumannii*. Consistent with these findings, a recently developed clinical chelator, endowed with extremely high affinity for iron, showed no significant anti-biofilm activity in *A. baumannii*. Thus, while iron metabolism continues to represent a promising target for *A. baumannii* inhibition, the impressive iron withholding capacity of this bacterium should be taken into account for future development of chelation-based antimicrobial and anti-biofilm therapies.

## Supplementary Materials

Supplementary materials can be accessed at: http://www.mdpi.com/2076-0817/3/3/704/s1.

## Supplementary Files

Supplementary File 1
