# Supplementary material for: Iron and Acinetobacter baumannii Biofilm Formation
Source: Pathogens. 2014 Aug 18;3(3):704–19. doi: 10.3390/pathogens3030704 (PMC4243436; doi:10.3390/pathogens3030704)
Supplement: Supplementary File 1 [file pathogens-03-00704-s001.pdf]

Supplementary Material

Table S1. Strains used in this study.

Strain (Alternative Designation)	Origin (Sample) [a]	Year of Isolation	Country	Antibiotic Susceptibility	Sequence Group	Reference
Km1008-06	VET (dog pus surgical wound)	2004–2009	Switzerland	MDR	1(ICL II)	5
OD474-06	VET (cat liver biopsy)	2004–2009	Switzerland		NV	5
Km693-07	VET (dog bronchial aspirate)	2004–2009	Switzerland	MDR	2(ICL I)	5
Km323-09	VET (dog eye swab)	2004–2009	Switzerland	MDR	1(ICL II)	5
Km456-05	VET (cat urine)	2004–2009	Switzerland		2(ICL I)	5
Km1245-05	VET (horse pus wound)	2004–2009	Switzerland		2(ICL I)	5
Km765-04	VET (dog pericardial effusion)	2004–2009	Switzerland		1(ICL II)	5
ACI_0638 (DVL5022)	VET (intravenous horse catheter)	2000–2008	Belgium	MDR	3(ICL III)	44
ACI_0639 (DVL4982)	VET (intravenous horse catheter)	2000–2008	Belgium	MDR	1(ICL II)	44
ACI_0644 (DVL5007)	VET (intravenous horse catheter)	2000–2008	Belgium		1(ICL II)	44
5966	VET (cat urine)	2000–2008	Germany		1(ICL II)	45
132	VET (dog pericardium)	2000–2008	Germany	MDR	2(ICL I)	45
4255	VET (dog abscess)	2000–2008	Germany	MDR	1(ICL II)	45
3204	VET (horse tendon)	2000–2008	Germany	MDR	1(ICL II)	45
P1697	VET (cat urine)	2000–2008	Germany		1(ICL II)	45
1782	VET (dog urine)	2000–2008	Germany	MDR	1(ICL II)	45
172	VET (dog urine)	2000–2008	Germany	MDR	1(ICL II)	45
P1959	VET (cat thorax)	2000–2008	Germany	MDR	1(ICL II)	45
4297	VET (dog wound)	2000–2008	Germany	MDR	3(ICL III)	45
196-1	VET (cat urine)	2000–2008	Germany	MDR	3(ICL III)	45
1231	VET (cat urine)	2000–2008	Germany	MDR	2(ICL I)	45
P869	VET (horse cervix)	2000–2008	Germany	MDR	3(ICL III)	45
760	VET (dog blood)	2000–2008	Germany	MDR	1(ICL II)	45
1173	VET (dog wound)	2000–2008	Germany	MDR	1(ICL II)	45
1356	VET (dog fistula)	2000–2008	Germany	MDR	3(ICL III)	45
3061	VET (cow udder)	2000–2008	Germany		14	45
4199	VET (dog vagina)	2000–2008	Germany		8	45
A60	HCL [a] (blood or respiratory secretions)	2001–2004	Argentina	MDR	1(ICL II)	21,47
A369	HCL [a] (blood or respiratory secretions)	2001–2004	Spain	MDR	1(ICL II)	21,47
A372	HCL [a] (blood or respiratory secretions)	2001–2004	Greece	MDR	2(ICL I)	21,47
A377	HCL [a] (blood or respiratory secretions)	2001–2004	Germany	MDR	3(ICL III)	21,47
A390	HCL [a] (blood or respiratory secretions)	2001–2004	Bulgaria	MDR	2(ICL I)	21,47
A402	HCL [a] (blood or respiratory secretions)	2001–2004	Taiwan	MDR	1(ICL II)	21,47
A437	HCL [a] (blood or respiratory secretions)	2001–2004	Croatia	MDR	2(ICL I)	21,47
A440	HCL [a] (blood or respiratory secretions)	2001–2004	Bulgaria	MDR	2(ICL I)	21,47
A458	HCL [a] (blood or respiratory secretions)	2001–2004	Estonia	MDR	2(ICL I)	21,47
A472	HCL [a] (blood or respiratory secretions)	2001–2004	Poland	MDR	2(ICL I)	21,47
A491	HCL [a] (blood or respiratory secretions)	2001–2004	India	MDR	1(ICL II)	21,47
50C	HCL (respiratory secretions)	2004	Italy	MDR	1(ICL II)	49

Table S1. *Cont.*

Strain (Alternative Designation)	Origin (Sample) [a]	Year of Isolation	Country	Antibiotic Susceptibility	Sequence Group	Reference
139L	HCL (respiratory secretions)	2007	Italy	MDR	1(ICL II)	49
175P	HCL (wound swab)	2008	Italy	MDR	1(ICL II)	49
141M	HCL (respiratory secretions)	2007	Italy	MDR	1(ICL II)	49
82D	HCL (wound swab)	2004	Italy	MDR	2(ICL I)	49
3130	HCL (blood)	2004	Lebanon	MDR	2(ICL I)	50
3889	HCL (bronchial aspirate)	2005	Greece	MDR	1(ICL II)	51
4025	HCL (bronchial aspirate)	2005	Lebanon	MDR	3(ICL III)	52
3868	HCL (bronchial aspirate)	2003	Turkey	MDR	12	51
3871	HCL (bronchial aspirate)	2003	Turkey	MDR	5	53
3865	HCL (blood)	2005	Turkey	MDR	12	53
3909	HCL (bronchial aspirate)	2007	Italy	MDR	8	54
ATCC 17978	HCL (respiratory secretions)	1951	France		NV	29
AYE	HCL (urine)	2001	France	MDR	2(ICL I)	31
ACICU	HCL (cerebrospinal fluid)	2005	Italy	MDR	1(ICL II)	32
RUH 5875	HCL (blood)	1997	Netherlands	MDR,	3 (ICL III)	34
ATCC 17978 (pMP220::P*basA*)	HCL (respiratory secretions)	1951	France		NV	26,29

Abbreviations: VET, veterinary; HCL, human clinical; MDR, strain resistant to three or more antimicrobial classes [55]; NV, new sequence group type variant; ICL, international clonal lineage. [a] human clinical isolates obtained from ARPAC project (A60–A491) derived from either respiratory secretion or blood, without specification from the sender laboratory.

Additional references for the Supplementary Material

49. D'Arezzo, S.; Capone, A.; Petrosillo, N.; Visca, P.; GRAB; Ballardini, M.; Bartolini, S.; Bordi, E.; di Stefano, A.; Galiè, M.; *et al.* Epidemic multidrug-resistant *Acinetobacter baumannii* related to European clonal types I and II in Rome (Italy). *Clin. Microbiol. Infect.* **2009**, *15*, 347–357.
50. Zarrilli, R.; Vitale, D.; di Popolo, A.; Bagattini, M.; Daoud, Z.; Khan, A.U.; Afif, C.; Triassi, M. A plasmid-borne blaOXA-58 gene confers imipenem resistance to *Acinetobacter baumannii* isolates from a Lebanese hospital. *Antimicrob. Agents Chemother.* **2008**, *52*, 4115–4120.
51. Pournaras, S.; Markogiannakis, A.; Ikonomidis, A.; Kondyli, L.; Bethimouti, K.; Maniatis, A.N.; Legakis, N.J.; Tsakris, A. Outbreak of multiple clones of imipenem-resistant *Acinetobacter baumannii* isolates expressing OXA-58 carbapenemase in an intensive care unit. *J. Antimicrob. Chemother.* **2006**, *57*, 557–561.
52. Di Popolo, A.; Giannouli, M.; Triassi, M.; Brisse, S.; Zarrilli, R. Molecular epidemiological investigation of multidrug-resistant *Acinetobacter baumannii* strains in four Mediterranean countries with a multilocus sequence typing scheme. *Clin. Microbiol. Infect.* **2011**, *17*, 197–201.
53. Vahaboglu, H.; Budak, F.; Kasap, M.; Gacar, G.; Torol, S.; Karadenizli, A.; Kolayli, F.; Eroglu, C. High prevalence of OXA-51-type class D beta-lactamases among ceftazidime- resistant clinical isolates of Acinetobacter spp.: Co-existence with OXA-58 in multiple centres. *J. Antimicrob. Chemother.* **2006**, *58*, 537–542.

54. Giannouli, M.; Cuccurullo, S.; Crivaro, V.; di Popolo, A.; Bernardo, M.; Tomasone, F.; Amato, G.; Brisse, S.; Triassi, M.; Utili, R.; *et al.* Molecular epidemiology of multidrug- resistant *Acinetobacter baumannii* in a tertiary care hospital in Naples, Italy, shows the emergence of a novel epidemic clone. *J. Clin. Microbiol.* **2010**, *48*, 1223–1230.
55. Magiorakos, A.P.; Srinivasan, A.; Carey, R.B.; Carmeli, Y.; Falagas, M.E.; Giske, C.G.; Harbarth, S.; Hindler, J.F.; Kahlmeter, G.; Olsson-Liljequist, B.; *et al.* Multidrug-resistant, extensively drug-resistant and pandrug-resistant bacteria: An international expert proposal for interim standard definitions for acquired resistance. *Clin. Microbiol. Infect.* **2012**, *18*, 268–281.

© 2014 by the authors; licensee MDPI, Basel, Switzerland. This article is an open access article distributed under the terms and conditions of the Creative Commons Attribution license (http://creativecommons.org/licenses/by/3.0/).
